# Age Moderates the Link between Epilepsy and Self-Rated Health (SRH)

**DOI:** 10.3390/jcm11206175

**Published:** 2022-10-19

**Authors:** Weixi Kang

**Affiliations:** UK DRI Care Research and Technology Centre, Department of Brain Sciences, Imperial College London, London W12 0BZ, UK; weixi20kang@gmail.com; Tel.: +44-73-980-44185

**Keywords:** epilepsy, self-rated health, age

## Abstract

Epilepsy is one of the most severe neurological diseases that affect people around the globe. Self-rated health (SRH) refers to one’s subjective evaluation of their own health and is associated with various outcomes such as morbidity and mortality. Thus, understanding the association between epilepsy and SRH is of great importance. Moreover, SRH generally decreases with age. The aim of the current study is to test whether age moderates the link between epilepsy and SRH. The current study used a hierarchical regression and three multiple regressions to analyze the associations between epilepsy and SRH in 529 epilepsy patients and 46,978 healthy controls from the United Kingdom. The current study found that age significantly moderates the association between epilepsy and SRH. Specifically, epilepsy status was negatively related to SRH in young people (*b* = −0.69, *p* < 0.001, 95% C.I. [−0.84, −0.54]), more strongly in middle-aged adults (*b* = −0.81, *p* < 0.001, 95% C.I. [−0.95, −0.66]), and most strongly in older adults (*b* = −0.89, *p* < 0.001, 95% C.I. [−1.09, −0.69]). The current study may imply that older adults need more attention in terms of their SRH, which is closely associated with outcomes. Clinicians and health professionals should come up with ways that improve SRH in people with epilepsy, especially for older adults with epilepsy.

## 1. Introduction

To better identify and track the burden of epilepsy in communities, it is important to develop and enhance the infrastructure and capacity for surveillance and epidemiologic studies of people with epilepsy and is as a key priority in doing so [1]. Burdens induced by epilepsy include both social and personal dimensions, which could cause direct and indirect costs such as health care costs, impaired well-being, and can negatively affect physical and mental health (see [2]).

Self-rated health (SRH) refers to one’s subjective evaluation of their own health and has been widely used as an indicator of general health in healthy research at the population level, which integrates biological, functional, mental, and social aspects of a person [3]. Although SRH is non-specific, it has good predictive validity, as demonstrated by its associations with various health-related conditions such as stroke, lung disease, arthritis, functional impairment, cardiovascular disease, depression [4], and outcomes such as morbidity and mortality [5]. There are few studies that have looked at SRH in epilepsy patients. For instance, Kobau et al. [2] found that adults with active epilepsy and with a history of epilepsy were more likely to report fair or poor health in a cohort from the United States. Kang (2022) found that epilepsy patients are characterized by poorer SRH compared to healthy controls [6].

Age is also a consistent predictor of SRH, given that objective health decreases with age. Indeed, studies have found that SRH declines with age (e.g., [7,8]). These declines could be also explained by age-related health conditions. For instance, cardiovascular disease is more prevalent in older people compared to younger people [9]. Several studies suggested that health conditions are the most critical factors for formulating the subjective health evaluation change across age, independent of gender [10].

Thus, one would suspect that age moderates the link between epilepsy and SRH, and understanding how epilepsy is associated with SRH has a profound meaning in terms of understanding the outcomes, as SRH is predictive of various health-related outcomes. The aim of the current study is to investigate how age moderates the associations between epilepsy and SRH.

## 2. Methods

### 2.1. Data

This study extracted data from Understanding Society: the UK Household Longitudinal Study (UKHLS), which has been collecting annual information from the original sample of UK households since 1991 (when it was previously known as The British Household Panel Study (BHPS). This dataset is publicly available at https://www.understandingsociety.ac.uk/documentation/mainstage, accessed on 10 September 2022. All data collections have been approved by the University of Essex Ethical Committees. Participants received informed consent before participating in these studies. Data were used from Wave 1, which was collected between 2009 and 2010 [11]. There were 529 epilepsy patients and 46,978 healthy controls in the current study. Descriptive statistics can be found in Table 1.

### 2.2. Measures

#### 2.2.1. Epilepsy

Self-reported epilepsy is a valid measure to identify epilepsy at a population level (e.g., [12]). Participants answered the question “Has a doctor or other health professional ever told you that you have any of these conditions? Epilepsy.” to indicate if they have epilepsy.

#### 2.2.2. SRH

Participants responded to the question, “In general, would you say your health is...” using a 5-point scale ranging from 1 (excellent) to 5 (very poor). The reliability of this single measurement of subjective health is moderate (e.g., [13]). SRH was reverse coded, so now 1 = very poor and 5 = excellent.

#### 2.2.3. Demographics Variable

Demographic variables include age, sex (male vs. female), monthly income, highest educational qualification (college vs. under college), and marital status (married vs. not currently married).

#### 2.2.4. Analysis

A hierarchical regression model [14] with demographic variables including age, sex, monthly income, highest educational qualification, marital status, epilepsy status, and epilepsy status by age interaction was taken into the model as predictors to predict SRH. Participants were then grouped into three groups based on their age, including young (16 and 35), middle-aged (35 to 55), and older people (above 55). Finally, three multiple regressions were used by taking demographic variables including age, sex, monthly income, highest educational qualification, and marital status; these variables were taken into the model as predictors of SRH for young, middle-aged, and older people.

## 3. Results

**Figure 1:**
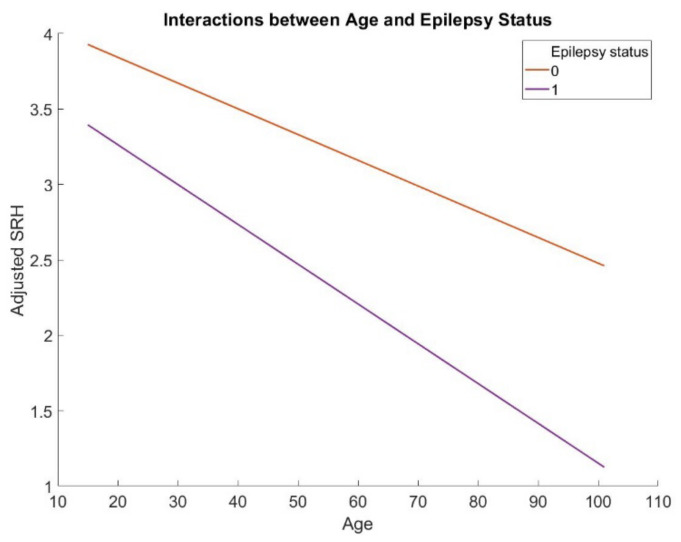
The interactions between age and epilepsy status in predicting SRH.

Descriptive statistics can be found in Table 1. The current study found that age significantly moderates the association between epilepsy and SRH (*b* = −0.009, *p* < 0.01, 95% C.I. [−0.015, −0.004]) according to the hierarchical regression [14]. Specifically, epilepsy status was negatively related to SRH in young people (*b* = −0.69, *p* < 0.001, 95% C.I. [−0.84, −0.54]), more strongly in middle-aged adults (*b* = −0.81, *p* < 0.001, 95% C.I. [−0.95, −0.66]), and most strongly in older adults (*b* = −0.89, *p* < 0.001, 95% C.I. [−1.09, −0.69]). The full results are presented in Table 2 and visualized in Figure 1.

## 4. Discussion

The aim of the current study was to investigate whether age moderates the link between epilepsy and SRH. By using a hierarchical regression and three multiple regressions on data from 529 epilepsy patients and 46,978 healthy controls, the current study found that age significantly moderates the association between epilepsy and SRH. Specifically, epilepsy status was negatively related to SRH in young people, more strongly in middle-aged adults, and most strongly in older adults.

The findings that epilepsy status is negatively associated with SRH across age groups seem to be consistent with previous studies (e.g., [2,6]). Indeed, epilepsy patients face a lot of challenges in their daily life including poor health, reduced physical activities, higher inflammation rate, more disability, and more risky behaviors [15,16,17,18]. Second, perceived limitations in social and emotional support can also lead to poor SRH, given that SRH is not only a reflection of objective health but also incorporates psychosocial aspects of a person. Third, neurological factors have adverse impacts on the SRH in people with epilepsy [19] as well. These challenges may in turn explain the poorer SRH in the current study.

The main finding of the current study was that age significantly moderates the association between epilepsy and SRH. Indeed, previous studies have found that SRH declines with age (e.g., [7,8]). Young people with a diagnosis of epilepsy had poorer SRH compared to healthy controls. However, this effect was larger in older participants. Older adults with declined health may be more sensitive to their diagnosis of epilepsy and may be more impeded by their epilepsy, which may then result in poorer SRH. Older adults with a diagnosis of epilepsy may also have awareness of their impaired physical health, which strengthens the link between epilepsy and SRH.

Despite the strengths of the current study, including a large data set and well-controlled sociodemographic characteristics, there are some limitations of the current study. First, the current study relied on self-reported data, which cannot avoid self-reporting bias. Future studies should confirm epilepsy status with medical diagnosis. Second, the current study is cross-sectional, which cannot establish causality. Future studies should use longitudinal approaches to establish casualties if possible. Third, the current study focused on participants from the United Kingdom, which may make it hard to generalize the current findings to other countries and cultures. Future studies should test how age may moderate the associations between epilepsy and SRH in various countries and cultures. Finally, unmeasured variables may contribute to the results of the current study, such as the frequency of seizures, discrimination, and stigmas. Future studies should control for these confounding variables.

## 5. Conclusions

Taken together, the current study found that age moderates the link between epilepsy and SRH, with older adults having the strongest negative association between epilepsy and SRH. The current study may imply that older adults need more attention in terms of their SRH, which is closely associated with outcomes. Clinicians and health professionals should come up with ways that improve SRH in people with epilepsy, especially for older adults with epilepsy. Specifically, social participation should be encouraged, as participation in the community salon was associated with improvements in SRH over time [20]. Moreover, eHealth tools can also be useful for encouraging healthy behaviors and improving SRH [21]. Moreover, as demonstrated by the results from the current study, obtaining at least a college degree and being married are also associated with better SRH. Moreover, one study has found that being involved in vigorous or moderate physical activities and frequently consuming legumes and eggs is associated with better SRH [22]. 

## Figures and Tables

**Table 1:** Descriptive statistics of demographic characteristics, epilepsy status, and SRH (self-rated health).

	Healthy Controls		Epilepsy Patients	
	Mean	S.D.	Mean	S.D.
Age	45.96	18.16	43.83	16.29
Monthly income	1221.67	1331.02	1042.10	957.65
SRH	3.40	1.15	2.58	1.21
	N	%	N	%
**Sex**				
Male	16,805	43.36	231	43.67
Female	22,105	56.32	298	56.33
**Highest educational qualification**				
Below college	33,811	71.97	434	82.04
College	13,167	28.03	95	17.96
**Legal marital status**				
Single	23,128	49.23	305	57.66
Married	23,850	50.77	224	42.34

**Table 2:** The regression coefficient (*b*) for the demographics and epilepsy status with the total explained variances (R^2^) for young, middle-aged, and older people. All numbers were rounded up to two digits. *** *p* < 0.001.

	Young	Middle-Aged	Older
Age	−0.02 ***	−0.02 ***	−0.01 ***
Sex	−0.16 ***	0.04 ***	0.15 ***
Monthly income	0.00 ***	0.00 ***	0.00 ***
Highest educational qualification	0.30 ***	0.33 ***	0.42 ***
Marital status	0.14 ***	0.24 ***	0.21 ***
Epilepsy status	−0.69 ***	−0.81 ***	−0.89 ***
R^2^	0.0385	0.0732	0.0596

## Data Availability

Publicly available datasets were analyzed in this study. This data can be found here: https://www.understandingsociety.ac.uk (accessed on 21 September 2022).

## References

[1] Epilepsy Foundation (2003). Living Well with Epilepsy II.

[2] Kobau R., Zahran H., Grant D., Thurman D.J., Price P.H., Zack M.M. (2007). Prevalence of active epilepsy and health-related quality of life among adults with self-reported epilepsy in California: California Health Interview Survey 2003. Epilepsia.

[3] Wuorela M., Lavonius S., Salminen M., Vahlberg T., Viitanen M., Viikari L. (2020). Self-rated health and objective health status as predictors of all-cause mortality among older people: A prospective study with a 5-, 10-, and 27-year follow-up. BMC geriatrics.

[4] Dowd J.B., Zajacova A. (2007). Does the predictive power of self-rated health for subsequent mortality risk vary by socioeconomic status in the US?. Int. J. Epidemiol..

[5] Jylhä M. (2009). What is self-rated health and why does it predict mortality? Towards a unified conceptual model. Soc. Sci. Med..

[6] Kang (2022). Epilepsy Patients Have Poor Life Satisfaction and Self-rated Health (SRH): Findings from the United Kingdom. Front. Psychol..

[7] Andersen F.K., Christensen K., Frederiksen H. (2007). Self-rated health and age: A cross-sectional and longitudinal study of 11,000 Danes aged 45–102. Scand. J. Public Health.

[8] Zajacova A., Huzurbazar S., Todd M. (2017). Gender and the structure of self-rated health across the adult life span. Soc. Sci. Med..

[9] Rieker P.P., Bird C.E., Lang M.E., Bird C.E., Conrad P., Fremont A.M., Timmermans S. (2010). Understanding Gender and Health: Old Patterns, New Trends, and Future Directions. Handbook of Medical Sociology.

[10] Read J.N.G., Gorman B.K. (2010). Gender and health inequality. Annu. Rev. Sociol..

[11] University of Essex, Institute for Social and Economic Research (2022). Understanding Society: Waves 1-11, 2009–2020 and Harmonised BHPS: Waves 1-18, 1991–2009. [Data Collection].

[12] Brooks D.R., Avetisyan R., Jarrett K.M., Hanchate A., Shapiro G.D., Pugh M.J., Berlowitz D., Thurman D., Montouris G., Kazis L.E. (2012). Validation of self-reported epilepsy for purposes of community surveillance. Epilepsy Behav..

[13] Zajacova A., Dowd J.B. (2011). Reliability of self-rated health in US adults. Am. J. Epidemiol..

[14] Aiken L.S., West S.G., Reno R.R. (1991). Multiple Regression: Testing and Interpreting Interactions.

[15] Centers for Disease Control and Prevention (2008). Epilepsy surveillance among adults—19 states, Behavioral Risk Factor Surveillance System, 2005. MMWR.

[16] Centers for Disease Control and Prevention (2005). Prevalence of epilepsy and health-related quality of life and disability among adults with epilepsy—South Carolina, 2003 and 2004. MMWR.

[17] Layne Moore J., Elliot J.O., Lu B., Klatte E.T., Charyton C. (2009). Serious psychological distress among persons with epilepsy based on the 2005 California Health Interview Survey. Epilepsia.

[18] Leidy N.K., Elishauser A., Vickrey B., Means E., William M.K. (1999). Seizure frequency and the health-related quality of life of adults with epilepsy. Neurology.

[19] Hermann B., Jacoby A. (2009). The psychosocial impact of epilepsy in adults. Epilepsy Behav..

[20] Ichida Y., Hirai H., Kondo K., Kawachi I., Takeda T., Endo H. (2013). Does social participation improve self-rated health in the older population? A quasi-experimental intervention study. Soc. Sci. Med..

[21] Seiwert K.A., Butler L., Maynard D., Kinkade M., Nill D.T. (2022). Improving Adolescent Self-Rated Health Using a Multiple Health Behavior Change eHealth Intervention. Am. J. Health Educ..

[22] Abuladze L., Kunder N., Lang K., Vaask S. (2017). Associations between self-rated health and health behaviour among older adults in Estonia: A cross-sectional analysis. BMJ Open.

